# Suicide risk following Emergency Department presentation with self-harm varies by hospital

**DOI:** 10.23889/ijpds.v8i2.2237

**Published:** 2023-09-14

**Authors:** Siobhán Murphy, Dermot O'Reilly, Emma Ross, Aideen Maguire, Denise O'Hagan

**Affiliations:** 1 Queen's University, Belfast, United Kingdom; 2 Public Health Agency, Belfast, United Kingdom

## Objectives

A large proportion of those who die by suicide present to an Emergency Department (ED) with self-harm (SH) in the year before death. This study examines ‘does risk of death following ED presentation with SH vary according to hospital attended?’

## Method

The Northern Ireland Self-Harm Registry provided data on SH presentations to 12 ED departments in NI between 2012-2019. Linkage to health and mortality records provided follow up to December 2019. Cox proportional hazards regression models were employed to assess mortality risk following presentation with SH among 12 ED departments in NI.

## Results

Analysis of the 64,350 ED presentations for self-harm by 30,011 individuals confirmed a marked variation across EDs in proportion of patients receiving mental health assessment and likelihood of admission to general and psychiatric wards. There was a significant variation in suicide risk according to ED attended with the three-fold range between the lowest (HRadj 0.32 95%CIs 0.16, 0.67) and highest. These differences persisted even after adjustment for patient characteristics, variation in types of self-harm, and care management at the ED.

## Conclusion

Management of SH cases in the ED is important, however, it is the availability, access and level of engagement with, care in the community rather than the immediate care at EDs that is most critical for patients presenting to ED with self-harm.

